# Rural Appalachia Battling the Intersection of Two Crises: COVID-19 and Substance-Use Disorders

**DOI:** 10.13023/jah.0204.10

**Published:** 2020-09-01

**Authors:** Margaret Miller, Rebekah Rollston, Kate Beatty, Michael Meit

**Affiliations:** American Academy of Family Physicians; Cambridge Health Alliance and Harvard Medical School; East Tennessee State University

**Keywords:** Appalachia, substance use disorders, COVID-19, health disparities

## Abstract

During the COVID-19 pandemic, rural Appalachia is at great risk of unforeseen side effects including increased mortality from substance use disorders (SUDs). People living with SUDs are at increased risk for both exposure to and poor outcomes from COVID infection. The economic impacts of COVID-19 must also be considered. As rural Appalachia combats the substance use crisis amidst the COVID-19 pandemic, the geographic economic, health and social inequities within our region must be considered. As a national recovery is sought, we should reimagine federal policies that center the economic and public health of rural Appalachia addressing the two crises.

In the midst of the COVID-19 pandemic, rural Appalachia is at risk for previously unforeseen side effects from physical distancing needed to contain the virus, including increased mortality from substance-use disorders (SUDs). In 2015, drug overdose mortality rates in Appalachia were 65% higher than the national average, driven largely by opioid use. While rates had declined, drug overdose mortality rates in Appalachia remained 48% higher than the national average in 2018, the most recent year of data available.^1^ Since that time, early data from the Centers for Disease Control and Prevention (CDC) show rising drug overdose mortality rates nationally, and anecdotally, partners in Appalachia attribute those most recent trends to the impact of COVID-19.

People living with SUDs are at increased risk for both exposure to and poor outcomes from COVID infection.^2^ They experience homelessness and incarceration at greater rates than the general population, making physical distancing difficult or impossible, placing individuals at increased risk for COVID-19 exposure and transmission.^3^ Other risks associated with SUDs include poverty, housing and food insecurity, lack of access to health care, and complex chronic conditions, all of which complicate proper screening and treatment for COVID-19.^4^ Further, chronic use of high-dose opioids restricts breathing, which can lead to chronic respiratory disease, increasing the risk for COVID-19 morbidity and mortality.^2^ Methamphetamine use also places individuals at higher risk due to blood vessel restriction, pulmonary vessel disease, and pulmonary hypertension.^2^

Even as individuals are at higher risk of overdose and COVID-19 mortality due to SUD, access to treatment services have been disrupted, including the vitally important medication-assisted treatment (MAT) for opioid-use disorder (OUD). MAT includes methadone, buprenorphine, and naltrexone. These substances are generally dispensed in-person in the context of a biopsychosocial clinic visit. Due to physical distancing measures, the federal government has relaxed restrictions, allowing providers to dispense 14- or 28-day prescriptions to patients who are deemed stable.^5^ Although these relaxed restrictions allow many patients with OUD to stay safely sheltered-in-place, some patients are still required to report to methadone clinics daily, or buprenorphine clinics weekly, to obtain their prescription. This requirement puts high-risk patients at greater risk for COVID exposure and transmission or risk of relapse and overdose if they are not able to access MAT. The use of telehealth services for MAT has significantly increased, thereby allowing many patients to stay safely sheltered-in-place while also continuing biopsychosocial treatment for addiction.^6^

Programs like Self-Management and Recovery Training (SMART Recovery), Alcoholics Anonymous (AA), Narcotics Anonymous (NA), and other faith-based programs are key for patients in recovery. Many of these programs have shifted to virtual programming throughout the COVID pandemic in order to promote community support for recovery. For example, the Kentucky Opioid Response Effort has compiled a list of virtual meetings for those in recovery.^7^ Such virtual programming may prove helpful for geographically isolated residents far beyond the COVID pandemic. Despite these new virtual opportunities, there is concern that some patients may have difficulty participating due to lack of access to reliable Internet or phone services.^8^

Additionally, syringe-service programs (SSPs)—community-based programs that provide access to and disposal of sterile syringes and injection equipment, vaccination, testing for infectious diseases, and naloxone distribution—have been negatively affected throughout the pandemic.^9^ Although considered essential public health services by the CDC,^9^,^10^ one study found that 25% of SSPs have closed at least one site since the start of the pandemic.^11^

The economic impacts of COVID-19 must also be considered. Based on data from the Great Recession, which strongly correlated unemployment with suicide and drug overdose deaths, the Well Being Trust and Robert Graham Center for Policy Studies in Family Medicine and Primary Care found that approximately 75,000 Americans could die due to drug or alcohol misuse or suicide as a result of the COVID-19 pandemic.^12^ This poses a significant concern due to loss of work and local economic decline for rural Appalachian residents.

As rural Appalachia combats the substance use crisis amidst the COVID-19 pandemic, we must reflect on the geographic, economic, health, and social inequities within our region. It is vital that as we seek a national recovery that results in a stronger America, we reimagine federal policies that center the economic and public health of rural Appalachia.^13^
